# Strong modulation of plasmons in Graphene with the use of an Inverted pyramid array diffraction grating

**DOI:** 10.1038/srep27550

**Published:** 2016-06-09

**Authors:** N. Matthaiakakis, H. Mizuta, M. D. B. Charlton

**Affiliations:** 1 Department of Electronics and Computer Science, University of Southampton, Southampton, SO17 1BJ, United Kingdom; 2 School of Materials Science, Japan Advanced Institute of Science and Technology, Ishikawa 923-1292, Japan

## Abstract

An optical device configuration allowing efficient electrical tuning of surface plasmon wavelength and absorption in a suspended/conformal graphene film is reported. An underlying 2-dimensional array of inverted rectangular pyramids greatly enhances optical coupling to the graphene film. In contrast to devices utilising 1D grating or Kretchman prism coupling configurations, both s and p polarization can excite plasmons due to symmetry of the grating structure. Additionally, the excited high frequency plasmon mode has a wavelength independent of incident photon angle allowing multidirectional coupling. By combining analytical methods with Rigorous Coupled-Wave Analysis, absorption of plasmons is mapped over near infrared spectral range as a function of chemical potential. Strong control over both plasmon wavelength and strength is provided by an ionic gel gate configuration. 0.04eV change in chemical potential increases plasmon energy by 0.05 eV shifting plasmon wavelength towards the visible, and providing enhancement in plasmon absorption. Most importantly, plasmon excitation can be dynamically switched off by lowering the chemical potential and moving from the intra-band to the inter-band transition region. Ability to electrically tune plasmon properties can be utilized in applications such as on-chip light modulation, photonic logic gates, optical interconnect and sensing applications.

Graphene is a flat monolayer of carbon atoms tightly packed in a two-dimensional honeycomb lattice. Although graphene was only recently discovered it has attracted a lot of attention as it combines remarkable electronic, photonic and mechanical properties^1^,^2^,^3^,^4^,^5^,^6^. In terms of photonic properties graphene has demonstrated high quantum efficiency for light matter interactions, strong optical nonlinearity, high optical damage threshold, and plasmons with high confinement and long propagation distances^7^,^8^,^9^. The reason graphene has attracted a large amount of interest in the photonics community is the linear dispersion of its Dirac electrons^8^ in combination with the ability to strongly alter its electronic, and optical properties through dynamic electrostatic modulation of its chemical potential by applying a gate voltage^5^.

Several methods have been implemented in order to couple and dynamically control plasmons in graphene. 1D trench based silicon diffraction gratings have been used as a wave vector/phase matching component between incident photons and plasmons in graphene. This provides control over the plasmon frequency by altering the grating lattice constant or by applying a voltage which alters the chemical potential in graphene^10^,^11^. Graphene microstructures and nanostructures have also been studied in order to couple and control localized plasmons. Ribbons^11^,^12^,^13^, disks^14^,^15^,^16^,^17^, and ring structures^16^,^17^,^18^ have been investigated, demonstrating control of the plasmon frequency by electrostatic gating and structure geometry or size. In every case shifting the plasmon resonance to near infrared spectral range and towards visible has been very challenging. The highest plasmon excitation wavelengths in this spectral range have been achieved with the introduction of hybrid metal/graphene structures, typically metamaterials^19^,^20^, gold nanoantennas^21^,^22^, nanorods^23^, and bowties^24^, in direct contact with a graphene layer. In this case plasmons are excited in the metal nanostructures and tuning is achieved through strong electrical coupling with graphene.

Obtaining strong coupling and highly tunable plasmons in graphene up to near infrared and visible frequencies is a difficult but highly anticipated task. In this work an inverted pyramid array diffraction grating with an ionic gel^25^,^26^ gate setup is proposed as an efficient coupling method for plasmons in Graphene. A two-dimensional array of inverted pyramid pits forms a crossed diffraction grating functioning as a phase-matching component coupling incident photons to plasmons in the graphene layer positioned above the diffraction grating as the active plasmonic medium. Ionic gel is chosen as the gate dielectric due to its transparent nature and high capacitance values when compared to conventional high-k gate dielectrics. Modulation of graphene chemical potential is then achieved by applying a small voltage across the ion gel/Silicon substrate. The configuration is shown in Fig. 1.

Theoretical calculations as well as Rigorous coupled-wave-analysis simulations were undertaken demonstrating excitation of a plasmon mode, with excellent control of plasmon energy up to near infrared spectral range, using realistic chemical potential values for graphene. A plasmon energy shift from 0.65 eV to 0.84 eV when changing the chemical potential of graphene from 0.45 eV to 0.6 eV was achieved.

We find that the structure supports a high frequency odd vector parity mode originating from the multi-interface nature of the device. This supports higher plasmon frequencies than typical single interface modes. Optimization of geometrical parameters such as pyramid size and pitch length were undertaken in order to achieve high coupling efficiency and demonstrate the scalable nature of the device. Unlike graphene micro and nanostructures, that can only support bound localized plasmon excitations^12^,^13^,^14^,^15^,^16^,^17^,^18^ this setup supports propagating plasmons in a continuous graphene layer. Propagating modes have been reported to have higher field confinement in the surface normal when compared to localized modes^11^. Furthermore, unlike previously reported grating based devices^10^,^11^, plasmon excitation for both s and p polarization is possible due to the symmetrical nature of the grating structure.

In practice n-doping of the graphene layer in combination with careful choice of the electrochemical window of ionic liquids used in the ionic gel can provide chemical potentials above 0.6 eV. Thus operation of the device can be extended for larger plasmon energies allowing plasmon excitations in the visible region of the spectrum.

## Electrical tuning of isolated graphene layer refractive index with chemical potential

Carbon atoms have a total of six electrons out of which only four valence electrons can participate in bonds. The remaining two core electrons are strongly bound to the nucleus. In graphene carbon atoms are arranged in a honeycomb lattice formed by the sp^2^ bonds^5^. The p_z_ orbitals of the neighboring carbon atoms overlap, forming bonding and antibonding states and thus the π-bands of graphene^5^,^6^. The low energy band structure of graphene involves the π electrons. The bonding π states form the valence band, and the antibonding π^*^ states form the conduction band^6^. These states are orthogonal and as a result cross each other. Valance and conduction bands touch at six points known as the Dirac points^5^,^6^. Two of these points (known as the K and K’ points) are independent^6^. The unit cell of graphene contains two carbon atoms and its lattice can be viewed as two separate sub-lattices (A and B) that are formed by those atoms^6^. Because of the symmetry between the A and B sub-lattices the conduction and valence bands are degenerate at the K and K’ points and as a result the electronic bands have a linear dispersion^5^. For small energies (below ~1.5 eV) the band structure can be considered as two symmetric cones with the conduction and valence bands touching at the Dirac point^6^, which is where the chemical potential of graphene is located for undoped samples.

The position of the chemical potential can easily be shifted above or below the Dirac point, (thus altering the carrier concentration in the material) by applying a voltage. The carrier concentration in Graphene in relation with the applied voltage can be calculated as follows^27^,^28^:


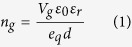


where *V*_*g*_ is the applied voltage, *ε*_*0*_ and *ε*_*r*_ the permittivity of vacuum and the relative permittivity of the substrate respectively, *e*_*q*_ the electron charge, and *d* the substrate thickness. Having obtained the carrier concentration of the system the Chemical potential can be calculated as^29^:


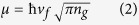


where ћ is the reduced Planck constant and, *v*_*f*_ Fermi velocity. To observe the change in optical behavior of graphene, conductivity is calculated as a function of wavelength taking into account the intraband and interband transitions. Chemical potential is obtained by use of the Kubo formula^30^:


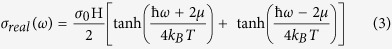



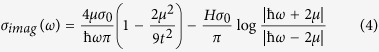



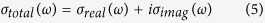


where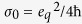, 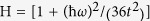, *T* the temperature, *t* the hopping parameter, and *ω* the angular frequency. By including a cubic term in the density of states this equation goes beyond the Dirac-cone approximation thus providing highly accurate results for high photon energies. Even though the response of the permittivity is essentially determined by valence and conduction electrons, the highly polarized environment originating from core electrons shouldn’t be ignored. The dielectric function including the contribution of the core electrons (background permittivity) can be expressed:


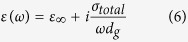


where 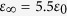 is the background permittivity^31^,^32^,^33^,^34^, and *d*_*g*_ graphene’s thickness (taken as 0.34 nm).

**Figure 2:**
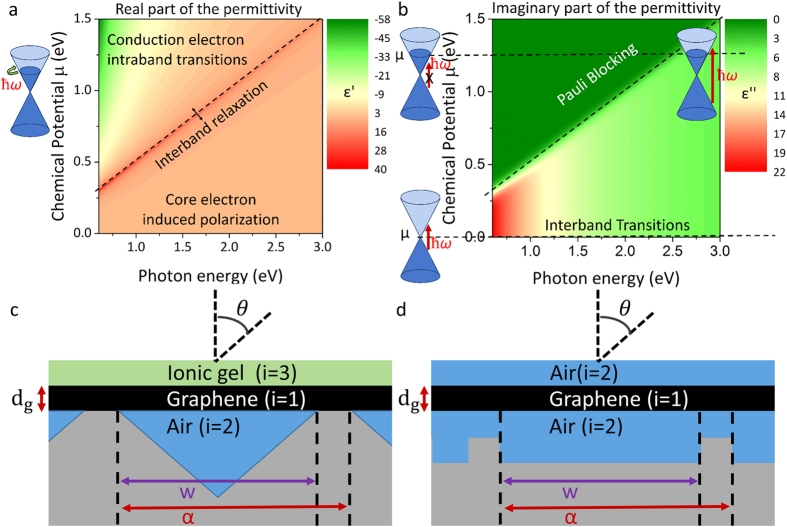
(**a)** Contour plot of the real part of the permittivity of graphene at visible and near infrared (NIR) wavelengths for a range of chemical potentials. Dirac cone diagrams are used to demonstrate the interactions responsible for the observed optical response and the dominant contributing mechanisms are labeled. The green arrows correspond to intraband transitions. (**b**) Contour plot of the imaginary part of the permittivity of graphene at visible and NIR wavelengths for a range of chemical potentials. The red arrows correspond to interband transitions. (**c**) Schematic of the multilayer setup and cross-section of the diffraction grating structure. (**d**) simplified device schematic used for theoretical calculations, the thickness of the two air layers is assumed as infinite when calculating the plasmon dispersion.

It should be mentioned that for wavelengths shorter than 410 nm (3.02 eV) an exciton-dominated peak with a maximum at 270 nm (4.6 eV) begins to appear^35^,^36^ and thus this model is no longer valid. Complex permittivity of graphene is plotted as a function of photon energy and chemical potential in Fig. 2a (*real*) and 2.b (*imaginary*) parts at a temperature *T* of 300 K and a hoping parameter *t* of 2.7 eV for photon energies between 0.4 eV and 3 eV and chemical potential 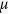 in the range 0 eV to 1.5 eV

For photon energies 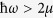 *imaginary* permittivity is positive, corresponding to energy loss for photons propagating through the material. This loss is due to absorption of photons by valence electrons participating in vertical interband transitions. In the same spectral region *real* permittivity has a stable value of 5.5 due to the polarization originating from the core electrons as seen in Fig. 2a. A sudden and severe change occurs in both *real* and *imaginary* permittivity at the limit where photon energy equals twice the chemical potential in graphene (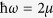). As conduction band states become occupied with electrons they become unavailable for transitions (Pauli blocking). As a result photons with energy less than twice the chemical potential (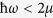

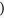
 cannot contribute to interband transitions and so losses experienced by light propagating through the material become low giving an almost zero value for *imaginary* permittivity.

Finally, for increasingly high chemical potential and when 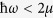, *real* permittivity becomes negative and thus graphene demonstrates plasma behavior. Due to the high chemical potential value, the conduction band has enough filled states, such that a large quantity of free electrons are provided which can flow in a similar way as in metals. In this spectral range Transverse Magnetic Surface plasmon polaritons can be supported due to the metallic behavior of graphene.

### Electrical tuning of simplified 3-layer Insulator/Graphene/Insulator system

The electrostatically controlled plasmon wave vector dispersion in graphene can be obtained by calculating the relation between the plasmon wave-vector and frequency. In multilayer systems of alternating dielectric/conducting materials each individual interface with a conducting layer can support Transverse Magnetic (TM) modes of bound surface plasmon polaritons^37^,^38^. When the separation distance (here *d*_*g*_) between the interfaces is smaller than the decay length 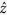 of individual TM modes (as is the case for mono/bi-layer graphene or very thin metal films) they begin to interact with each other resulting in coupled modes. Since graphene is a very thin conducting layer and the device under study in this work has dielectric materials both above (ionic gel) and below (air) the graphene layer, a multilayer assumption is used to obtain the plasmon dispersion (Fig. 2c). Taking into account only lowest order bound modes and TM modes that are non-oscillatory in the z-direction normal to the interfaces, plasmon wave-vector 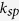 is implicitly related to frequency ω by the following equation: 37


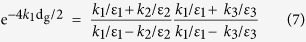


where 
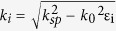
 is the component of the wave vector perpendicular to the interfaces for each distinct region (in this case i = 1, 2, 3 where i = 1 corresponds to the graphene layer) and 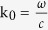 the wave-vector of the incident photons. For simplicity both dielectric materials will be assumed as infinitely thick and described by the permittivity of air (Fig. 2d). Since dielectrics above and below the graphene layer have equal permittivity values, equation (7) can be further reduced and the dispersion relation split as follows.


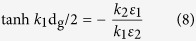



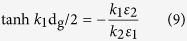


Equation (8) describes modes of odd vector parity (E_x_(z) component of electric field is odd, H_y_(z) component of magnetic field and E_z_(z) component of electric field are even functions) and equation (9) describes modes of even vector parity (E_x_(z) is even, H_y_(z) and E_z_(z) are odd functions)^37^.

**Figure 3:**
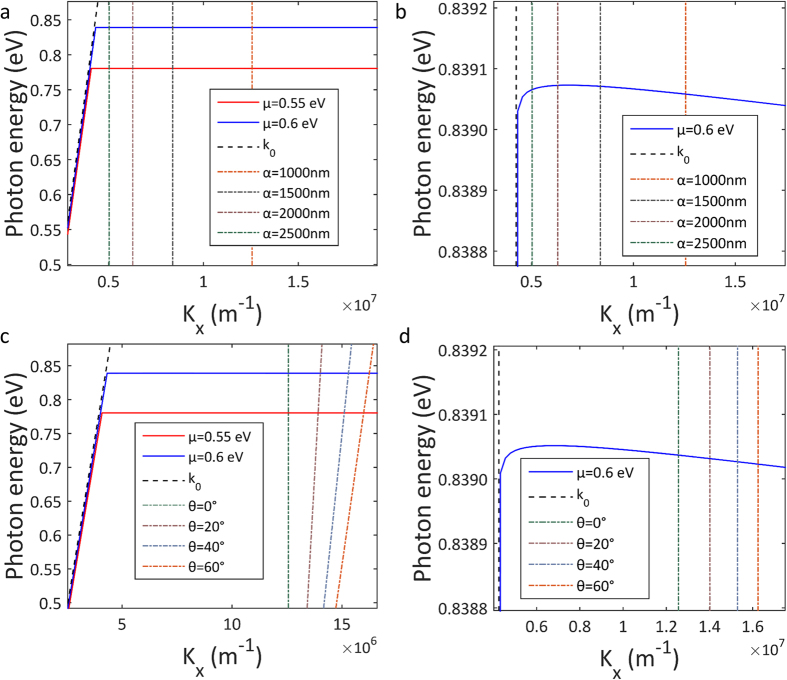
High frequency odd mode of the graphene plasmon dispersion is plotted as solid lines for a chemical potentials μ of 0.55 eV (red) and 0.6 eV (blue). The light line (k_0_) is plotted as a black dashed line and the dash-dot lines represent the diffracted photon wave-vector due to the diffraction grating. In (**a**) the diffracted photon lines are plotted for varying lattice constants α of the diffraction grating between 1000 nm and 2500 nm, (**b**) a zoomed in scale of photon energy axis showing the negative slope of the dispersion line for increasing wave-vectors. In (**c**) the diffracted photon lines are plotted for varying angles of incidence θ from 0 to 60 degrees, (**d**) a zoomed in scale of photon energy axis showing the negative slope of the dispersion line for increasing wave-vectors.

Figure 3a shows the dispersion of the high frequency odd modes obtained from equation (8) that are supported by this setup, for a Graphene layer with chemical potential of 0.55 eV and 0.6 eV. Strong modulation of the high frequency odd plasmon mode is observed with an increase of 0.05 eV in plasmon energy for just 0.04 eV increase in Graphene chemical potential. For Insulator-Conductor-Insulator systems such as this the odd mode can support excitations at frequencies higher than single interface (Insulator-Conductor) modes whose plasmon dispersion is described by 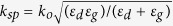, whereas the even mode supports lower excitation frequencies. Since the even mode has much lower excitation frequencies corresponding to wavelengths several hundreds of nm longer it is not further studied in this paper.

### Polarization independent phase-matching between incident photons and graphene plasmons by underlying 2D grating structure

Interaction between free carriers and the incident electromagnetic field results in wave vector of surface plasmon modes (ћk_sp_) being greater than that of incident photons (ћk_0_) for the same frequency. Because of the mismatch in wave-vector, surface plasmons cannot normally be generated and thus phase matching is required. In this device, phase change is achieved by the introduction of a 2-dimensional grating below the graphene in the form of an inverted pyramid array. A simple one-dimensional groove approximation (Fig. 2d) can be used to calculate the required lattice constants (or pitch) α of the diffraction grating. Phase matching between surface plasmons and incident photons takes place due to the increased wave-vector of the diffracted photons whenever


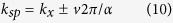


where grating order 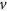 is an integer (1, 2, 3, …) and 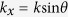 the in plane wave vector of impinging photons^37^.

Dispersion of the high frequency odd mode is plotted in Fig. 3a together with the diffracted photon wave-vector lines (solutions of equation 10) for diffraction grating lattice constants varying between 1000 nm and 2500 nm and a graphene chemical potential of 0.55 eV and 0.6 eV in graphene, with 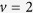. The dashed lines show the wave vector of directly diffracted photons. Points where the lines cross correspond to perfect phase matching conditions under which incident light couples to surface plasmon polaritons in graphene, *via* diffractive scattering from the underlying pyramid structure. Hence Fig. 3 visually displays phase matching conditions under electrical bias conditions for graphene.

Taking a closer look at the spectral region close to the plasmon excitation energy, Fig. 3b (which shows the same information as Fig. 3a but with highly zoomed y-scale) we see that the excitation frequency experiences a weak blue-shift (photon energy increases) as the diffraction grating lattice constant becomes longer. This blue-shift is expected for the high frequency odd mode^38^, and in this case is weak due to the extreme thinness of graphene and strong coupling between the top and bottom interface plasmon modes. This behaviour goes against typical single interface modes where longer grating lattice constants can induce reasonably strong redshifts in excitation frequency^11^.

Figure 3c,d show phase matching conditions for various angles of incidence for impinging photons. As expected larger angles of incidence result in an increase in diffracted photon wave vector (change in phase matching to the plasmon mode). However since the plasmon dispersion is almost flat, coupling can occur for a wide range of angles even up to 60° with no change of plasmon excitation frequency. This is extremely unusual for a grating coupler, and highly important because it means coupling is essentially non-directional. In practice, the structure will collect and couple incident light over a very wide range of angles of incidence, as is the case when light is tightly focused by a short focal lens. This is investigated in more depth in the next section.

### Optimization of practical device design by Rigorous coupled wave-analysis

Having observed some interesting (and unexpected) features in the graphene dispersion and grating phase match conditions arising from the simple analytical analysis (as shown visually in Fig. 3), we now move onto look at the properties of the phase matched solutions (points of intersection of the lines on Fig. 3) more carefully, by cross comparing to other methods of simulation.

Rigorous coupled wave-analysis (RCWA) is a highly efficient semi-analytical method for simulating electromagnetic behaviour of periodic structures. Electromagnetic fields and device geometries are represented by a sum of harmonic functions in Fourier space, and fields are solved in the form of a transmission line problem. RCWA simulations are particularly useful for graphene devices because (in contrast to FDTD or FEM methods) they don’t require solution of Maxwell’s equations over dense grids. Instead, structure is divided into uniform layers in the z direction and electromagnetic modes calculated by applying a layer by layer analytical solution which goes far beyond the simplified 3 layer system described in the previous section.

**Figure 4:**
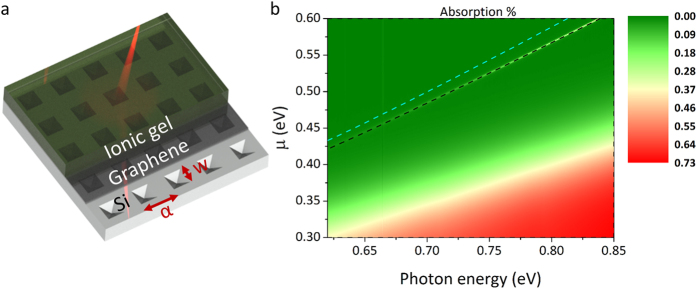
(**a**) 3D schematic of the simulated device under laser illumination demonstrating the pyramid structures, the graphene layer placed above them, and above the monolayer an ionic gel layer which is typically used to electrostatically modulate the chemical potential in graphene. Pitch (α) and pyramid side width (w) is also shown (**b**) RCWA simulation result demonstrating the large tuning range for plasmon excitations. Theoretical calculation results for the high frequency odd mode (black dashed lined) and single interface mode (cyan dashed line) can also be seen with the odd frequency mode having an excellent overlap with the plasmon absorption peak obtained from the simulation (white sharp line). On the lower part of the figure the onset of interband transitions can be seen.

In this section, relationship between geometrical parameters associated with the underlying inverted pyramid array diffraction grating (as indicated in Fig. 4a) and behaviour of coupled surface plasmons are investigated using RCWA simulations, for the purpose of optimising the geometry. Effect of geometry on plasmon energy, excitation efficiency (plasmon peak strength), and electrical modulation of plasmon frequency are investigated. Substrate permittivity is taken as that of Si.

A 50 nm thick ionic gel layer positioned above of graphene provides an alternative to high-k gate dielectrics, and provides a practical method to apply strong electrical modulation of graphene chemical potential. Parameters for the underlying diffractive pyramid structure correspond to those achieved by KOH etching of <100> silicon wafers. Graphene is then suspended above the inverted pyramid by (wet/dry) transfer process. Graphene is safely modelled as a 0.34 nm thick layer (since skin depth is significantly thicker in the spectral range of interest) with permittivity given by equation (6). Realistic thickness is chosen over thicker effective layers or a 2D sheet in order to have an accurate separation distance for the two interfaces.

### Electrical control of Plasmon absorption by adjustment of chemical potential

Figure 4b shows RCWA simulated plasmon absorption for underlying 1000 nm pitch (α)/500 nm side width (w) pyramid structure and a chemical potential in range of 0.3 eV to 0.6 eV. Solutions of the matched wave vectors coupled by the underlying structure calculated by the analytical method are superimposed as dashed lines for the high frequency odd mode (black) and the single interface mode (cyan) for 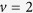.

An absorption peak is observed away from the interband transition region and at the location of the phase match frequency appearing as a sharp (white) line. As graphene chemical potential increases, the real part of permittivity becomes increasingly negative due to higher quantity of free carriers. Thus a blue-shift in plasmon excitation frequency is observed (dispersion line moves to larger energy), thereby confirming electrically tuneable plasmon absorption.

Looking more closely at the superimposed lines for the analytical model, for the single interface mode (cyan line) it predicts plasmon excitations at lower frequencies compared to RCWA method, whereas they are in excellent agreement to RCWA solutions for odd vector parity mode (black line). This provides proof that the excited mode is indeed a high frequency odd mode, and that the analytical model is actually very accurate. The higher plasmon frequencies supported by this mode when compared to plasmon excitations in single interface systems makes it ideal for scaling plasmons in graphene towards shorter wavelengths. In both cases strong modulation of plasmon excitation energy can be clearly observed with 0.04 eV shift in chemical potential resulting in ~0.05 eV shift in plasmon energy. Intensity of the absorption peak heavily depends on optical loss in graphene, mainly characterized by the imaginary part of permittivity, with higher losses corresponding to broader and lower peaks^11^. Plasmon excitation peaks become larger and narrower with increasing graphene chemical potential as a result of moving further away from the interband absorption region (observed as a broad absorption region at the lower part of the graph). We find that plasmon excitation can effectively be shut down by lowering graphene chemical potential. This is due to a change from interband to interband transition region of operation, and can occur over a broad wavelength range. This result is of great importance for applications as it provides a dynamic means of photonic switching which can be exploited for high density optical interconnects.

### Optimisation of 2D periodic structure geometry to maximize plasmon absorption and angular absorption range

The underlying 2D grating geometry can be scaled to shift the coupled graphene Plasmon frequency to match a broad range of incoming wavelengths and incidence angles, and also improve coupling efficiency. Plasmon absorption strength depends heavily on diffraction efficiency of the underlying grating structure used for phase matching. Improved diffraction efficiency increases coupling between incident light and graphene plasmons, resulting in higher intensity plasmon absorption peaks.

**Figure 5:**
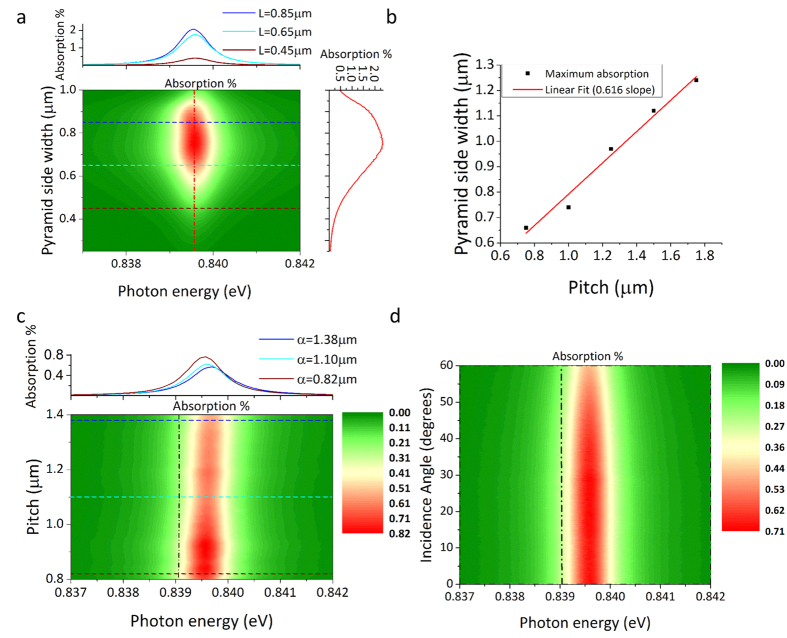
(**a**) The effect of pyramid size on plasmon absorption strength is demonstrated for pyramid side width ranging from 0.25 μm to 1 μm while maintaining an aspect ratio of 1.0. (**b**) The linear relation for pyramid size and pitch length is demonstrated for achieving highest plasmon excitation efficiency when scaling the device. (**c**) The effect of pitch length on plasmon energy and absorption strength is demonstrated, the brown, cyan and blue dashed lines over the contour plot correspond to the three plasmon peaks on the profile plot above. Theoretical calculation results are overlaid as the black dash-dot line. (**d**) The effect of incidence angle on plasmon energy and absorption strength is demonstrated. Theoretical calculation results are overlaid as the black dash-dot line.

Diffraction efficiency is related to density and size of diffractive structures. Optimization of size and spacing between inverted pyramids results in significantly improved diffraction efficiency. Effect of pyramid width (w) is investigated in Fig. 5a which shows RCWA simulations for fixed diffraction grating pitch of 1000 nm and chemical potential of 0.6 eV.

Plasmon absorption becomes stronger with increasing pyramid width, until the pyramid becomes approximately ¾ the size of the pitch where it begins decreasing again. Investigating this further, Fig. 5b reveals a linear relationship between pyramid size and grating pitch allowing prediction of maximum plasmon absorption. This is helpful when scaling the device for different applications. Plasmon energy (*frequency)* is found to be unaffected by pyramid size.

Diffracted photon k-vector is related to grating pitch (α) and so provides control of phase match frequency between incident photons and graphene plasmons. This is investigated in Fig. 5c, for pyramid width w = 500 nm, and graphene chemical potential of 0.6 eV. A small blue-shift of plasmon energy is observed with increasing pitch length (α), which is in agreement with theoretical expectations for the high frequency odd mode. This occurs because the odd parity mode dispersion has a negative relation between energy and wave-vector in this spectral region. Results calculated by the analytical method are again superimposed as the black dash-dot line.

The analytical calculations are found to be highly accurate agreeing with RCWA simulations within 0.001 eV (1 nm).

Virtually no shift in excitation energy is observed as a function of incidence angle (Fig. 5d) for a 1000 nm pitch/500 nm pyramid width grating structure, and 0.6 eV graphene chemical potential. This is also predicted by the analytical calculation as seen by the superimposed black dash-dot line. Wide angle wavelength independent absorption of incident light is very unusual and useful in practice. High efficiency coupling of incident light by short focal length high numerical aperture lens is predicted.

### Polarisation independent coupling

**Figure 6:**
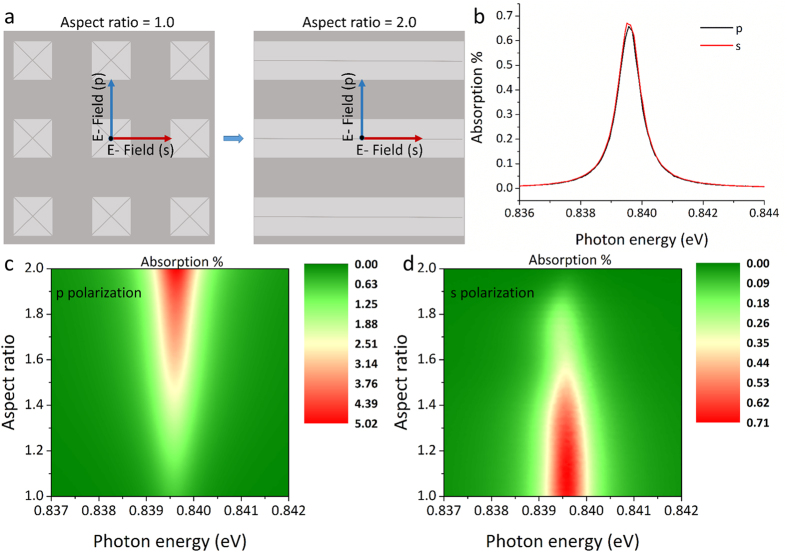
(**a**) Demonstration of the E field direction in respect to the diffraction grating for s and p polarization for gratings of 1.0 and 2.0 aspect ratio. In (**b**) plasmon excitation for a structure with an aspect ratio of 1.0 is compared for p and s polarization revealing identical absorption peaks. In (**c,d**) the effect of aspect ratio of the pyramid sides on plasmon excitation for s and p polarization can be seen where (**c**) corresponds to p polarization and (**d**) to s polarization.

Phase matching can only occur for surface plasmon polaritons propagating perpendicular to the diffraction features when incident photons are polarized in the same direction^39^. Unlike 1-Dimensional trench based gratings^10^,^11^, the symmetric pyramid structure diffracts both s and p polarizations with equal efficiency as seen in Fig. 6a,b.

Finally it is interesting to see what happens when the aspect ratio of the system changes and how this affects plasmon excitations for different incident photon polarizations. Increasing the width (w) of only one side of the pyramid (moving from square to rectangular structures) breaks the symmetry, thus an increase of coupling efficiency for the polarization satisfying excitations perpendicular to the direction of the extended feature is expected. At the same time a decrease of efficiency is expected for the other polarization. This can be observed in Fig. 6b–d where for the 1.0 aspect ratio there is no difference between s and p polarisations and the excited plasmons have the same absorption strength. Figures 6c,d reveal that when moving towards the 2.0 ratio, coupling due to s polarized light begins to decrease until it becomes 0 when the structure becomes a continuous trench, while coupling due to p polarized light becomes significantly stronger due to the extended diffraction structure in the direction favouring this polarization.

### Effect of dissipative losses

It has been reported that dissipative losses in graphene in the Terahertz^40^ and also in the Infrared and Optical frequencies^41^ can be significant thus hindering the potential of graphene as a plasmonic material. Even though for suspended graphene layers carrier mobility in excess of 200.000 cm^2^ V^−1^ s^−1^ has been demonstrated (by employing current induced heating in order to reduce impurities), these high mobility values are limited over a small range of carrier concentrations^42^. For unsuspended devices recently high mobility values have been demonstrated for CVD graphene by introducing hBN/graphene/hBN heterostructures on Si/O2, achieving mobility values comparable to exfoliated graphene (as high as 350.000 cm^2^ V^−1^ s^−1^ at low temperatures and above 50.000 cm^2^ V^−1^ s^−1^ at room temperature) but once again for larger carrier concentrations the scattering loss increases^43^. Ionic gels can achieve very high electrostatic doping levels in graphene at the expense of introducing strong carrier scattering. Typical carrier mobility for graphene devices with ionic gel gates ranges between 500 cm^2^ V^−1^ s^−1^ and 1200 cm^2 ^V^−1^ s^−1^ for unsuspended graphene layers^17^,^26^,^44^,^45^.

**Figure 7:**
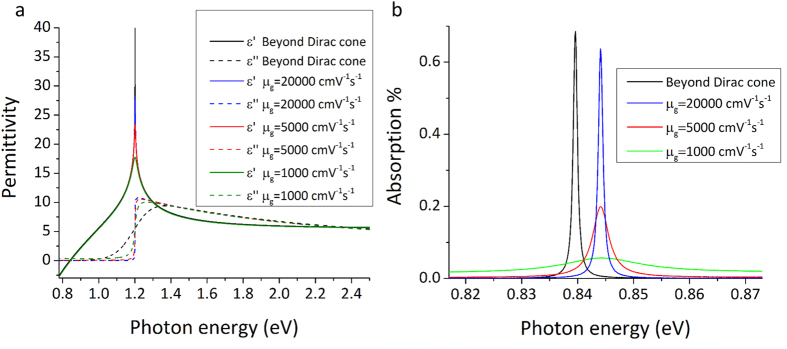
(**a**) Complex permittivity of graphene for different mobility rates at 0.6 eV chemical potential. The black lines represent results obtained when including a cubic term in the density of states (thus departing from the linear Dirac cone approximation) and the thermal broadening for interband transitions. (**b**) RCWA simulation results for different mobility values for a chemical potential of 0.6 eV. Decreasing carrier mobility results in shallower and broader absorption peaks. Black line corresponds to absorption spectra as obtained when modeling the graphene layer while including thermal broadening for interband transitions and the cubic term in the density of states.

Even though this study is a purely theoretical approach, it is important to investigate how the device performs when including experimentally obtained mobility values in the calculations. In this case simplified equations for the conductivity of graphene are used instead of equations (3) and (4):^46^


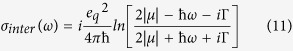



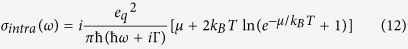


where the mobility is included through the phenomenological scattering rate 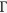. Unlike in equations (3) and (4) where a cubic term is included in the density of states, equations (11) and (12) do not go beyond the limitations of the Dirac cone approximation. A further simplification is the exclusion of thermal broadening for interband transitions. Nevertheless these simpler equations can accurately demonstrate the effect of dissipative losses in the absorption spectra when included in RCWA calculations by choosing different mobility rates. The resulting complex permittivity of the graphene layer for different mobility values μ_g_ is demonstrated in Fig. 7a in comparison with the result obtained through the use of equations (3) and (4).

By including the permittivity values for different mobility rates when modeling the graphene layer for the RCWA simulations the effect of dissipative loss can be observed in the absorption spectra as seen in Fig. 7b. Due to the linear Dirac cone assumption and thus the exclusion of the cubic term in the density of states the position of the absorption peak is slightly blue shifted when compared to the result obtained through equations (3) and (4). Since equations (3) and (4) go beyond the limitations of the Dirac cone approximation, the position of the absorption spectra obtained from them is expected to be more accurate, nevertheless the difference is very small. As seen in the simulation results, absorption spectra is largely depended on losses in graphene and the peaks become significantly shallower and broader with decreasing mobility. Dissipative losses can thus have a very negative effect on plasmon absorption and can be a very significant issue when designing real world applications. Therefore, it is crucial that care is taken to improve the mobility in the graphene layer by reducing layer damage, impurities, and substrate interactions, while maintaining strong electrostatic control over the chemical potential. Further research is required for providing easily transferred CVD graphene samples with high mobility values as well as effective gating methods that do not introduce strong scattering in the graphene layer.

## Conclusion

In conclusion the reported optical device configuration shows strong electrical modulation of surface plasmon energy and absorption intensity. The underlying 2-dimensional array of inverted pyramids is highly efficient for coupling photons to the graphene film supporting excitation of plasmons equally for both s and p polarizations due to the structure symmetry. By optimizing the diffraction efficiency of the underlying structure significant increase in plasmon absorption intensity was observed. The device provides highly efficient dynamic modulation of the plasmon energy operating over a very large spectral range and up to the near infrared for currently easily achievable graphene chemical potentials. Assuming higher chemical potentials operation of the device can be extended to the visible spectrum as is. Furthermore, plasmon excitation can be effectively shut down by lowering the chemical potential providing dynamic means of photonic switching which can be exploited for high density optical interconnects. Plasmon absorption was also found to be highly unaffected by angle of incidence thus providing the possibility of exciting plasmons on the monolayer with the use of high numerical aperture lenses. Finally an analytical model assuming a multilayer setup was presented with excellent agreement to the RCWA simulation results for the high frequency odd plasmon mode providing an easy way to estimate the operation and design of new devices. Over-all the device can be utilized in a large number of possible applications including sensors, photonic logic gates, optical interconnects and modulators.

## Additional Information

**How to cite this article**: Matthaiakakis, N. *et al*. Strong modulation of plasmons in Graphene with the use of an Inverted pyramid array diffraction grating. *Sci. Rep*. **6**, 27550; doi: 10.1038/srep27550 (2016).

## Figures and Tables

**Figure 1:**
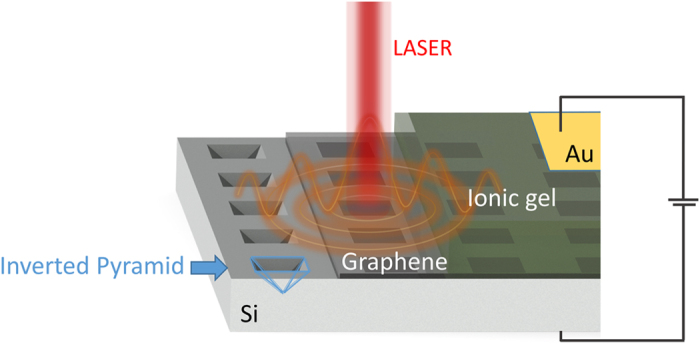
Schematic of the device setup showing the graphene layer sandwiched between the diffraction grating and the ionic gel that is used as the gate dielectric. When the device is illuminated by a laser beam photons are diffracted in the inverted pyramid pits and then couple with plasmons in graphene.
